# Arnold–Chiari malformation is associated with increased likelihood of a procedure in idiopathic intracranial hypertension

**DOI:** 10.3389/fopht.2025.1668498

**Published:** 2025-10-28

**Authors:** Jiawen Ma, Philip Nguyen, Jade Lee, Chaturica Athukorala, Kate Reid

**Affiliations:** 1 Department of Medical Imaging, The Canberra Hospital, Canberra, ACT, Australia; 2 ANU College of Health and Medicine, Australian National University, Canberra, ACT, Australia

**Keywords:** cerebellar tonsillar ectopia, Arnold Chiari malformation, idiopathic intracranial hypertension, cerebral venous hypertension, transverse sinus stenting

## Abstract

**Background:**

Approximately 18% of patients with idiopathic intracranial hypertension (IIH) prove medically refractory, and eventually require a procedure to manage their condition. An additional 2% have fulminant disease and require an immediate procedure to preserve vision. Identifying a neuroradiologic marker to stratify IIH patients more likely to require a procedure would assist clinical management and outcomes. Here, the authors explore whether cerebellar tonsillar position is such a marker.

**Methods:**

This was a retrospective, single-center cohort study of 180 consecutive patients with IIH in Canberra, Australia. Patient outcomes were classified as procedural intervention versus medical therapy alone. Cerebellar tonsillar position was measured relative to the foramen magnum to the nearest millimeter, as defined by the McRae line. The tonsillar position was classified as at or above the line, or lowered if below. Subsets of lowering were defined as “descent” (<3mm below), “ectopia” (3–5 mm below), or “Arnold–Chiari malformation” (>5 mm below). Two observers independently assessed the patients’ initial neuroimaging, and a random sample of 20 was also assessed by a more senior radiologist. Measurement precision was assessed using intraclass correlation coefficients, and patient outcome was analyzed against tonsillar position using univariable penalized logistic regression modeling.

**Results:**

91% of patients were female. The tonsils were at or above the McRae line in 36% (65/180) of patients and lowered in 64% (115/180). In 7%, lowering amounted to Arnold–Chiari malformation (13/180). Among those who underwent a procedure, the average tonsillar position was 1.94mm below the foramen magnum, whereas in those not requiring a procedure, it was 0.80mm. Across the whole cohort, the average tonsillar position was 1.0mm below the McRae line. The position of the cerebellar tonsils across the whole cohort was only mildly correlated with the likelihood of a procedural outcome (p = 0.04). However, true Arnold–Chiari malformation was strongly associated with procedural intervention at 46% (6/13), compared with 18% (30/167) in those without the malformation, with a relative risk of 2.57 and risk difference of 28% (odds ratio 5.15, 95% CI 1.45–18.52, p = 0.01). There was high concordance between the two observers’ measurements (0.89, 95% CI 0.81–0.93) and with the measurements obtained by the senior radiologist (0.97, 95% CI 0.93–0.99).

**Conclusion:**

The presence of Arnold–Chiari malformation in patients with idiopathic intracranial hypertension is associated with an increased likelihood of requiring a procedure. Moreover, independent of procedural outcome, cerebellar tonsillar lowering occurs in IIH patients so frequently that it should be further investigated as a potential neuroradiologic “soft sign” of the disease.

## Introduction

In recent years, there have been major advances in understanding cerebral venous hypertension as a key contributor to idiopathic intracranial hypertension (IIH). Where a significant pressure gradient exists across a stenosis of one or both transverse sinuses, the resultant venous hypertension can be effectively treated by stenting the stenosis (1). Stenting is highly effective in reversing papilledema in the 18% of IIH patients who prove medically refractory over time to pharmacologic therapy and weight loss (2).

Given that such a significant proportion of patients require procedural intervention, it would be helpful to identify them at presentation. This would allow better discussion at diagnosis of the treatment likely to be required—thereby mitigating against patients discontinuing IIH medication due to side effects (3) and/or being lost to follow-up (4). Approximately 2% of IIH patients with fulminant disease require immediate cerebrospinal fluid (CSF) diversion (5), but these patients are readily identified from the initial ophthalmic examination findings, especially the visual threat evident in the visual fields. However, it remains difficult to predict which patients with non-fulminant disease will ultimately fail medical therapy in the long term. At presentation, neither the severity of papilledema nor the lumbar puncture (LP) opening pressure correlates with the degree of cerebral venous hypertension observed at subsequent catheter venography (2).

The literature reports that cerebellar tonsillar ectopia or frank Arnold–Chiari malformation (ACM) may co-exist with IIH (6). Here, the authors explore whether lowering of the cerebellar tonsils below the foramen magnum might predict which IIH patients will ultimately require a procedure, either stenting or CSF diversion.

## Method

### Patient population

Retrospective data were collected for 180 consecutive IIH patients who presented to Canberra Hospital between 2009 and 2022. Patients were diagnosed with IIH according to the modified Dandy criteria (7), and all were confirmed by the senior author as having clinical evidence of IIH. Patients were initially suspected of having IIH on the basis of typical symptomatology and/or disc swelling. The latter was assessed by fundoscopy, Heidelberg optical coherence tomography (OCT) of the retinal nerve fiber layer (RNFL), and Humphrey automated visual fields. OCT autofluorescence was used to exclude optic nerve head drusen. Other causes of pseudotumor cerebri, such as a space-occupying lesion or cerebral venous sinus thrombosis, were excluded at initial neuroimaging. LP was routinely employed, both to measure the opening pressure and to ensure that cerebrospinal fluid (CSF) constituents were normal. Patients were monitored once on treatment to confirm the reduction of papilledema. Where diagnostic uncertainty persisted, confirmation of an IIH diagnosis was obtained with intracranial catheter venography and manometry via the femoral vein. Radiographic “soft signs” of IIH were not used to make the diagnosis, and, in particular, the position of the cerebellar tonsils was not considered as a diagnostic criterion at presentation.

CSF opening pressure data were available for 142 patients, with the remainder having undergone LP elsewhere. The method of LP (image-guided or not, prone vs. lateral recumbent) and body mass index (BMI) data were unavailable.

The large majority of patients receiving a procedure underwent stenting of their transverse sinus stenosis. They were deemed medically refractory on the grounds of ongoing visual threat (persisting papilledema or onset of optic atrophy on maximal medical therapy), intolerance of maximal medical therapy due to side effects, or disabling headache. These patients were subsequently investigated for a “stentable” transverse sinus stenosis (gradient >8 mmHg) (2) using intracranial catheter venography and manometry. Selection for CSF diversion rather than stenting was based on pediatric age, patient preference, or fulminant IIH. Again, cerebellar tonsillar position was not considered. No patients underwent both stenting and CSF diversion.

### Neuroimaging

Patients had received either computed tomography (CT) of the brain and venogram and/or magnetic resonance imaging (MRI) of the brain and venogram, available on the Canberra Hospital Medical Imaging Picture Archiving and Communication System (PACS). The procedural outcome for each patient was known to the senior author at the commencement of the study, but was withheld from the other authors until image analysis was complete.

Imaging was analyzed to determine the cerebellar tonsillar position to the nearest millimeter. The tonsillar position was classified as *at or above* the McRae line or *lowered* if below it. The degree of lowering was further categorized as *descent* (<3mm below), *ectopia* (3–5 mm below), or *Arnold–Chiari malformation* (>5 mm below) (8, 9).

The authors did not analyze patient neuroimaging for the “soft signs” of IIH because of their lack of specificity. Numerous imaging signs are associated with IIH (10, 11), including tortuosity of the optic nerve, dilation of its sheath, a partially or completely empty sella, and slit-like ventricles. Lesser-known soft signs include widening of the foramen ovale (12) and temporal encephalocele (13). However, these signs have not been validated as predictors of the need for procedural intervention or as specific indicators of IIH (14).

**Figure 1:**
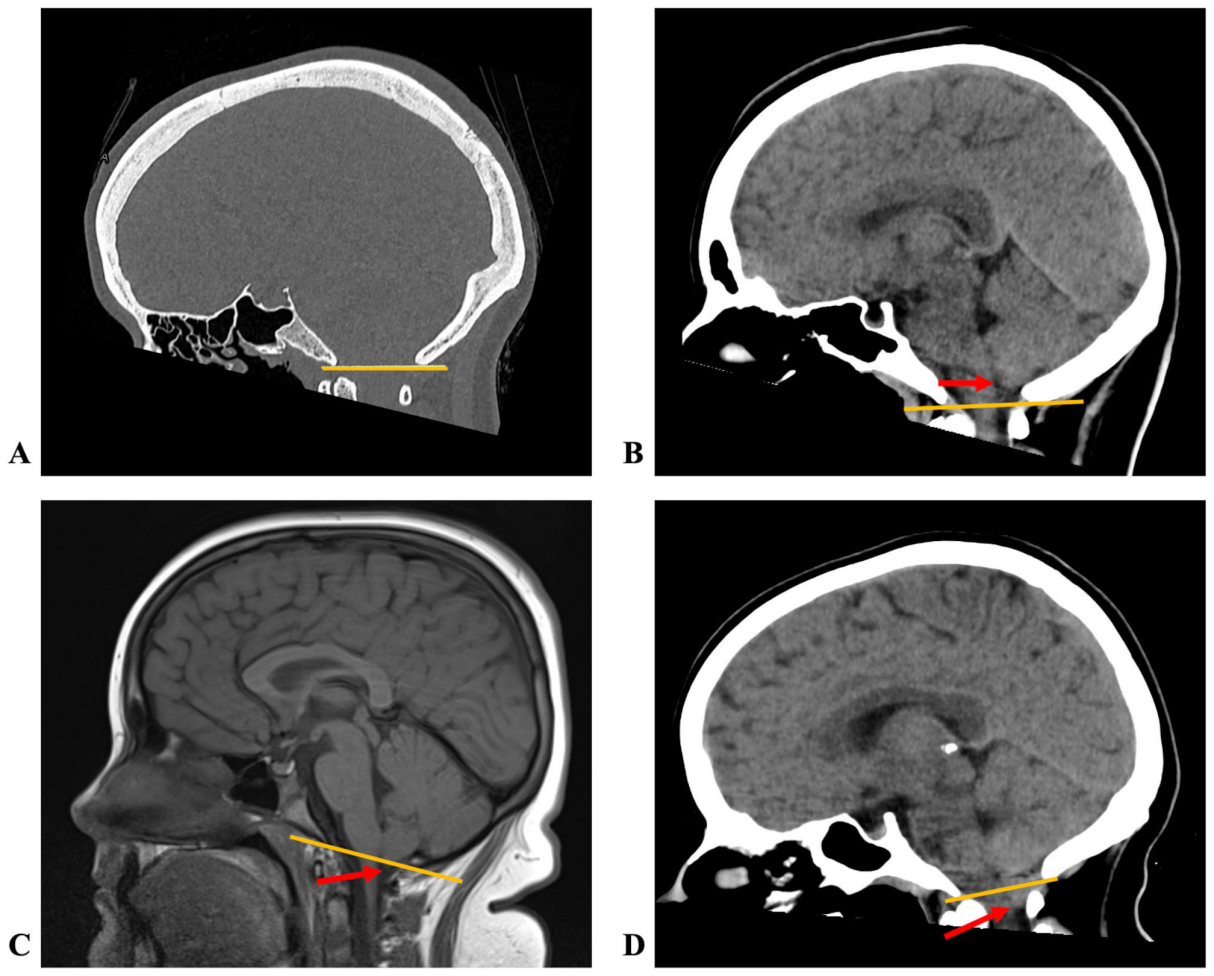
Sagittal images showing the McRae line in yellow; red arrows indicate the tip of the cerebellar tonsil. **(A)** CT bone window; **(B)** CT with the cerebellar tonsil above the line; **(C)** T1-weighted MRI with the cerebellar tonsil at the line; **(D)** CT with cerebellar tonsillar ectopia.

All cerebellar tonsillar measurements were performed on the AGFA Healthcare Enterprise Imaging Platform using a standardized approach. Multiplanar reformatting was performed on 0.5 mm–slice CT brain studies to ensure measurements were made in the true sagittal plane. Sagittal T1-weighted images were used for cerebellar tonsillar position measurement in the MRI studies. The McRae line was drawn from the basion to the opisthion of the occipital bone on CT bone reformats or MRI T1-weighted images (**Figure 1**). The perpendicular distance from the McRae line to the lowest cerebellar tonsillar apex was recorded to the nearest millimeter.

A consultant radiologist then measured a random selection of 20 patients to verify the results obtained independently by one senior and one junior radiology trainee. The observers were blinded to each other’s measurements and to the procedural outcome until final analysis.

All statistical analyses were performed using R 4.4.1 (R Core Team, Vienna, Austria). Inter-rater reliability for the trainees was assessed by calculating the intraclass correlation coefficient (ICC), using a two-way mixed-effects model with reliability estimation for a single rater. Subsequently, logistic regression analysis was performed to determine the correlation between cerebellar tonsillar position and the likelihood of procedural intervention, using the average of both observers’ measurements.

Subgroup analysis was conducted for the four tonsillar position groups (at/above the McRae line, descent, ectopia, and frank ACM). Owing to the low prevalence of ACM, Firth’s penalized logistic regression was used for analysis, and profile penalized likelihood confidence intervals were calculated. A simple multivariable logistic regression was performed with the additional covariate of CSF opening pressure—a potential key confounder—in 142 patients to assess its effect on the outcome. The effect sizes were reported.

Differences in tonsillar position between the CT- and MRI-measured groups were analyzed using the Mann–Whitney–Wilcoxon test. Modality-specific reliability was assessed within the regression model and through exclusion sensitivity analysis with the larger CT-measured cohort.

## Results

**Figure 2:**
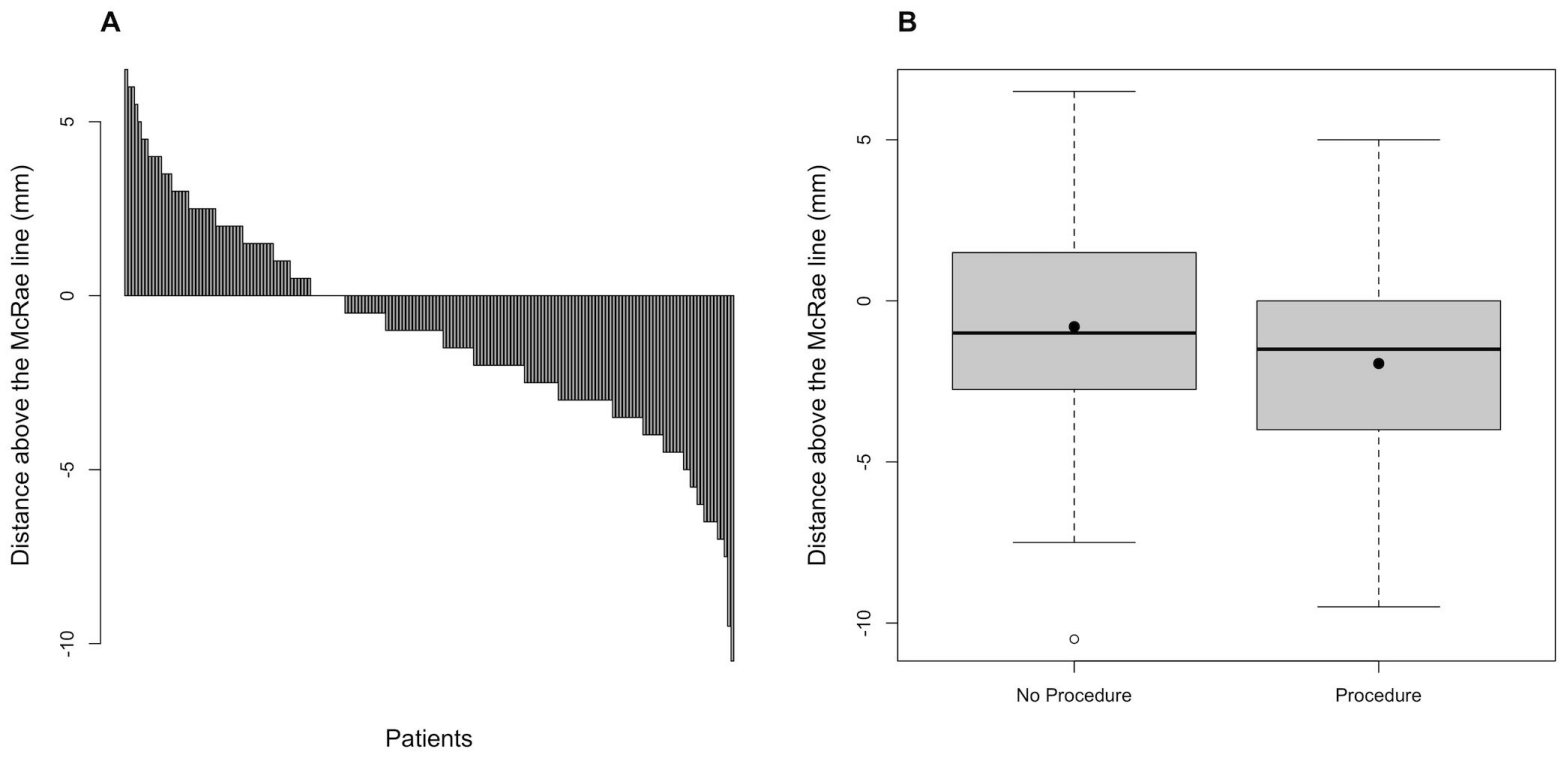
**(A)** Bar-plot distribution of cerebellar tonsillar position in the IIH cohort; **(B)** Box-plot comparing cerebellar tonsillar position in patients who did not receive a procedure with those who did. Means are shown as black dots, and medians as horizontal lines.

Data from 180 patients were included in the final analysis. 91% (164/180) of patients were female. 36% (65/180) had tonsils at or above the McRae line, and 64% (115/180) had lowered tonsils. 35% (63/180) had tonsillar descent, 22% (39/180) had tonsillar ectopia, and 7% (13/180) had frank Chiari malformation type I (**Figure 2A**). 20% (36/180) of patients underwent a procedure. Of these 36 patients, 30 underwent transverse venous sinus stenting after a medically refractory course, four had ventricular CSF shunting, one underwent optic nerve sheath fenestration (ONSF), and one had a craniectomy.

Within the ACM procedural group of six patients, four were treated with a stent (67%), one with a shunt (17%), and one with a craniectomy (17%). In the non-ACM procedural group of 30 patients, 26 were treated with a stent (87%), three with a shunt (10%), and one with ONSF (3%).

The mean cerebellar tonsillar position for the procedural group was 1.94mm below the McRae line, compared with 0.80mm below for the non-procedural group (odds ratio [OR] 1.14, 95% CI 1.00–1.30, p = 0.04). In the procedural group, the tonsillar position ranged from 5mm above the McRae line to 9.5mm below. In the non-procedural group, the range was 6.5mm above to 7.5mm below, with a single 10.5 mm outlier (**Figure 2B**).

Upon subgroup analysis (**Table 1**), the odds of IIH patients with ACM requiring a procedure were 5.15 times that of patients without ACM (p = 0.01, 95% CI 1.45–18.52). 46% (6/13) of IIH patients with ACM required a procedure, compared with 18% (30/167) in the non-ACM group. This equates to a relative risk of 2.57 and an absolute risk difference of 28% for procedural intervention in ACM.

**Table 1:** Subgroup analysis of patients categorized according to cerebellar tonsillar position.

Subgroup	Procedure rate	Relative risk	Odds ratio (95% CI)	p-value
Chiari 1 malformation (>5mm below McRae line)	6/13 (46%)	2.57 (0.46/0.18)	5.15 (1.45 – 18.52)	p = 0.01
Tonsillar ectopia (3–5 mm below McRae line)	10/39 (26%)	1.39 (0.26/0.18)	2.12 (0.79 – 5.76)	p = 0.14
Tonsillar descent (<3mm below McRae line)	11/63 (17%)	0.82 (0.17/0.21)	1.30 (0.51 – 3.40)	p = 0.58
No tonsillar lowering (≥0 mm above McRae line)	9/65 (16%)	0.59 (0.16/0.23)	–	–
Sex (Male)	6/16 (39%)	2.05 (0.39/0.18)	2.78 (0.89 – 8.68)	p = 0.07

By comparison, there was no significant correlation between a procedural outcome and either ectopia (p = 0.14) or descent (p = 0.58). The procedure rate for patients with ectopia was 26% (10/39) and for those with descent 17% (11/63).

Males were not more predisposed to a procedural outcome (p = 0.07).

CSF opening pressure data were available for 142 patients. These data showed similar characteristics to the full cohort in gender distribution (92% female), procedural rate (20%), and tonsillar position (38% at/above the McRae line, 32% descent, 23% ectopia, and 7% ACM). CSF opening pressure was not correlated with a procedural outcome (p = 0.27) and did not alter the effect of tonsillar position (p = 0.04).

### Data reliability

There was high concordance among all imaging observers. The two-way intraclass correlation coefficient between the two primary observers measured 0.89 (95% CI 0.81–0.93). The intraclass correlation coefficient of a subset of 20 randomly selected patients assessed by a third consultant radiologist measured 0.97 (95% CI 0.93–0.99).

Tonsillar position was measured on CT in 91% (164/180) of patients and on MRI in the remaining 9% (16/180). There was no significant difference in tonsillar position between the MRI and CT cohorts (p = 0.43). The imaging modality used for tonsillar measurement did not show significance when included as a covariate in the logistic regression model (p = 0.68), and it did not reduce the association of ACM with a procedural outcome (p = 0.01). In the CT-only cohort, there was a slight reduction in the strength of the association between ACM and a procedural outcome (p = 0.04).

## Discussion

### Arnold–Chiari malformation predicts procedural intervention in IIH

Identification at the initial diagnostic workup of IIH patients likely to need a procedure would prove a valuable adjunct to current management algorithms. Since ACM is associated with an increased likelihood of a procedure in IIH in this study, the authors believe that ACM should be specifically sought in the neuroimaging obtained at first presentation. ACM serves as a *prognostic* sign that can usefully be added to the *diagnostic* imaging findings attributable to IIH itself. By comparison, tonsillar lowering of lesser severity than ACM was not a statistically significant predictor of a procedural outcome in our cohort.

**Figure 3:**
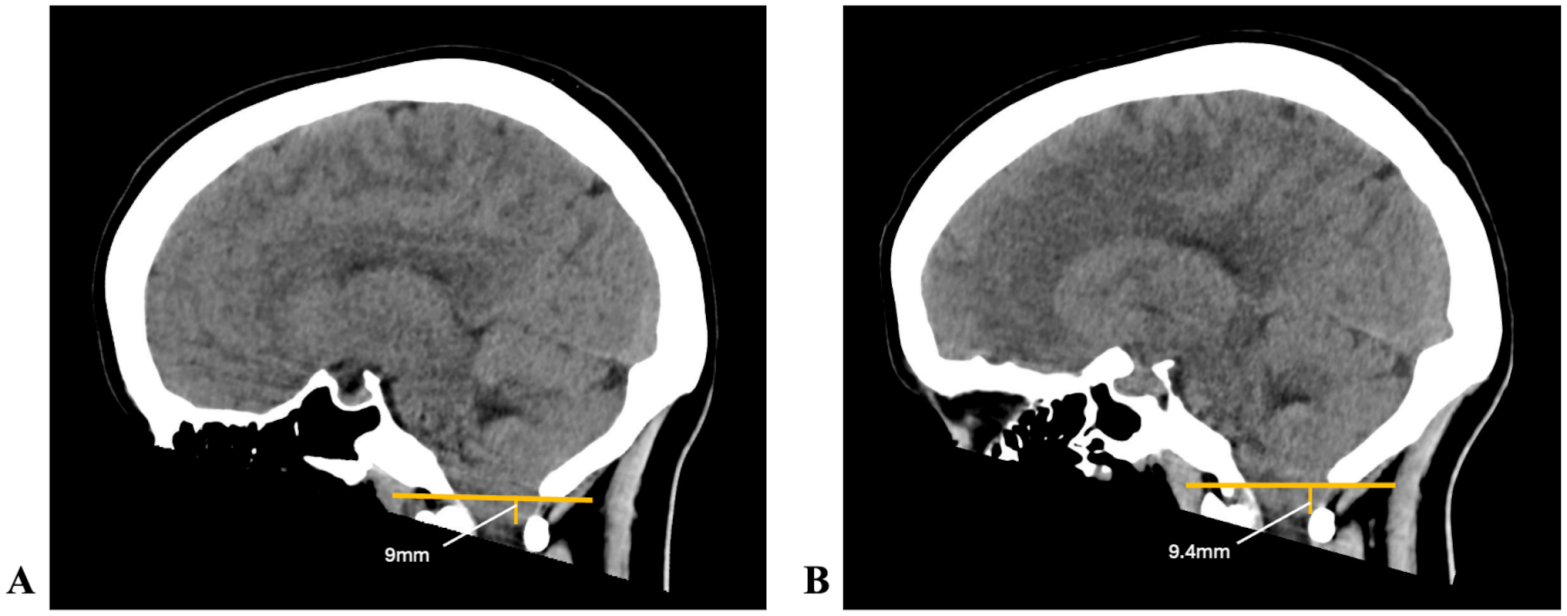
Cerebellar tonsillar position below the McRae line in a patient with Chiari malformation before and after ventriculoperitoneal shunting. **(A)** Before: 9mm; **(B)** After: 9.4 mm.

The co-existence of Chiari malformation and IIH is a well-known but poorly understood phenomenon, and numerous case reports have detailed the need for eventual CSF diversion after limited success with conventional Chiari treatment via suboccipital decompression (15). Some authors have proposed a Chiari–Pseudotumor Cerebri Syndrome (16). Aiken et al. (17) suggested that severe tonsillar lowering in IIH is acquired rather than congenital, hypothesizing that the anatomical finding is secondary to chronically increased intracranial pressure. However, there was no radiologic change in tonsillar position in one of our ACM patients after insertion of a ventriculoperitoneal shunt (**Figure 3**).

It may be that the presence of ACM predisposes to cerebral venous hypertension, which is not relieved by suboccipital decompression alone. The mechanism by which ACM elevates intracranial pressure does not appear to be simple obstructive hydrocephalus at the level of the foramen magnum, as there is no ventriculomegaly. Novel techniques, including artificial intelligence (AI), may in the future shed more light on the link between ACM and IIH (18).

### Cerebellar tonsillar lowering as an ancillary diagnostic indicator of IIH

64% (115/180) of our IIH study population had some degree of cerebellar tonsillar lowering below the McRae line, with 35% (63/180) showing descent, 22% (39/180) showing ectopia, and 7% (13/180) showing frank ACM. Ectopia and ACM were found in our patients at rates comparable to those in other studies of IIH cohorts (17, 19).

At 64%, tonsillar lowering in our IIH patients occurred with a prevalence far greater than the background 0.5 – 1% rate described in the sparse literature (9). Tonsillar lowering is not currently considered a neuro-radiological ‘soft sign’ of IIH, and the few studies that have examined this issue have reported conflicting results. Butros et al. (12) found no correlation between ‘low-lying cerebellar tonsils’ and IIH amongst their 92 patients. However, Aiken et al. (17) noted a correlation between lowered tonsillar position and IIH within their cohort of 87 patients.

Studies by Aboulezz et al. (20) and Barkovich et al. (21), which measured cerebellar tonsillar position on MRI in normal populations, did not comment on the prevalence of lowering. Nevertheless, they measured the average tonsillar position as 2.9mm and 1.0mm *above* the foramen magnum, respectively. Our total IIH population had an average tonsillar position of 1.0mm *below* the foramen magnum. Given these conflicting findings, further assessment of our study population against a control non-IIH cohort might establish tonsillar lowering as a soft neuroimaging sign of IIH—albeit not a prognostic one unless there is frank ACM.

### Data reliability

Both observers demonstrated extremely strong concordance, with an inter-rater reliability of 89%. Including an independently measured subset of patients assessed by a third observer, a consultant radiologist, increased the inter-rater reliability to 97%. These findings substantiate the robustness and simplicity of the methodology.

### Limitations

The greatest limitation of this study was the use of univariable analysis due to the relatively small cohort size. CSF opening pressure, a possible confounder, was available for only 79% of the total cohort and was included in a simple adjusted regression model, where it had only a minimal effect. Another potential confounder, BMI, was unavailable. The absence of complex multivariable modeling, primarily constrained by cohort size, may have led to under- or overestimation of the significance of cerebellar tonsillar position in predicting a procedural outcome.

Most patients received either CT or MRI of the brain at the initial workup. Potential measurement bias could therefore have occurred depending on the modality used to assess tonsillar position. After adjusting for measurement modality in the regression model, exclusion sensitivity analysis, and interaction tests, no confounding effect on the primary outcome was detected. However, the lack of simultaneous CT and MRI measurements within the same patients limited the robustness of reliability assessment. We noted that the McRae line was easier to define on CT scans, whereas the exact tip of the cerebellar tonsil was best appreciated on MRI (**Figure 1**).

Another important limitation of our analysis was that the primary outcome—procedural intervention—encompassed disparate procedures (venous stenting, CSF shunting, ONSF, and craniectomy), although these were heavily weighted toward stenting (83% of all procedures). However, our focus was on the need for any procedural intervention in IIH, irrespective of which pathophysiologic mechanism that procedure addressed. The low number of non-stent procedures precluded valid analysis of whether cerebellar tonsillar position in IIH predicts which specific type of procedural intervention would be required.

## Conclusion

ACM appears to be associated with an increased likelihood of an IIH patient requiring procedural intervention, although its low prevalence introduces statistical uncertainty in small cohort studies. Further research is needed to substantiate these findings. While tonsillar lowering of lesser degrees does not reliably predict the need for a procedure, it warrants additional investigation as a potential soft neuroimaging sign of IIH in this increasingly common disease.

## Data Availability

The raw data supporting the conclusions of this article will be made available by the authors upon request, without undue reservation.
